# Deletion of gE in Herpes Simplex Virus 1 Leads to Increased Extracellular Virus Production and Augmented Interferon Alpha Production by Peripheral Blood Mononuclear Cells

**DOI:** 10.3390/pathogens13121138

**Published:** 2024-12-23

**Authors:** Manon Claeys, Jonas Delva, Cedric Jacqmotte, Cliff Van Waesberghe, Herman W. Favoreel

**Affiliations:** Department of Translational Physiology, Infectiology and Public Health, Faculty of Veterinary Medicine, Ghent University, Salisburylaan 133, 9820 Merelbeke, Belgium; msclaeys.claeys@ugent.be (M.C.); jonas.delva@ugent.be (J.D.); cedric.jacqmotte@ugent.be (C.J.); cliff.vanwaesberghe@ugent.be (C.V.W.)

**Keywords:** HSV-1, glycoprotein E, type I interferon, peripheral blood mononuclear cells, plasmacytoid dendritic cell

## Abstract

Herpes simplex virus (HSV) in humans and pseudorabies virus (PRV) in pigs are both alphaherpesviruses. Plasmacytoid dendritic cells (pDCs) make part of the peripheral blood mononuclear cells (PBMCs) and are specialized in producing large amounts of antiviral type I interferon (IFN-I). IFN-I production by PBMCs in response to both HSV-1 and PRV can be virtually exclusively attributed to pDCs. Recently, we discovered that cells infected with gEnull PRV trigger increased production of IFNalpha by porcine PBMCs/pDCs compared with cells infected with wild-type (WT) PRV. This increased IFNalpha response correlates with increased extracellular virus production triggered by gEnull PRV compared with WT PRV. The gE protein and some of its currently described functions are conserved in different alphaherpesviruses, including PRV and HSV-1. In the current study, we report that cells infected with gEnull HSV-1 trigger increased IFNalpha production by human PBMCs and increased extracellular virus production compared with WT HSV-1. Hence, these recently described functions of PRV gE are conserved in HSV-1 gE. Since the increased extracellular virus production and IFNalpha response have also been reported for successful (gEnull) PRV vaccines, the current findings may have important consequences for the rational design of HSV vaccines.

## 1. Introduction

Herpes simplex virus and pseudorabies virus (PRV) belong to alphaherpesviruses, the largest subfamily of herpesviruses. HSV typically causes asymptomatic or mild infection with cold sores (mainly HSV-1) or genital lesions (mainly HSV-2) in immune competent humans. However, in immunodeficient individuals, infection may lead to severely aggravated disease, including herpes stromal keratitis, meningitis, or encephalitis [1]. Inadequate antiviral type I interferon (IFN-I) responses are one of the main underlying causes of aggravated HSV infection and disease [2]. Plasmacytoid dendritic cells (pDCs) are innate immune cells that are unique in their ability to produce and secrete massive amounts of IFN-I, up to 1000× more than any other cell type [3,4]. Importantly, both in vitro and in vivo, pDCs are the major source of IFN-I during stimulation or infection of peripheral blood mononuclear cells (PBMCs) by herpesviruses, indicating that pDCs play an important role in controlling herpesvirus infections [5,6,7]. In line with this, pDC depletion in mice has been associated with reduced survival upon systemic HSV infection [8]. Furthermore, human pDCs are important in the immune control of recurrent genital HSV infections [9], and, more recently, pDCs in the cornea of humans and mice are thought to play a critical role in preventing HSV-induced keratitis, the leading cause of human infectious blindness in the industrialized world [10].

To this day, no effective vaccines against HSV are available. An adaptive Th1 response is critical for a protective immune response against HSV infection [9,11]. Interestingly, via their production of IFN-I and other cytokines including IL-12, pDCs are central drivers of Th1 responses not only in humans but also in pigs [12,13,14]. Of note, highly effective attenuated vaccines have been developed for the porcine alphaherpesvirus PRV, in particular the cell-culture-adapted PRV Bartha vaccine strain [15]. We demonstrated that cells infected with Bartha PRV vaccine strains (or the Bucharest PRV strain) elicit strongly increased IFN-I responses by pDCs compared with cells infected with wild-type PRV strains [16]. This increased pDC response is to a substantial part caused by the deletion of the US8 gene in these PRV vaccine strains, which leads to increased production of extracellular virus particles that trigger increased IFN-I production by pDCs [16,17].

The viral US8 gene encodes the gE glycoprotein that is conserved in alphaherpesviruses. The aim of the current study was to assess whether the impact of the lack of gE on IFN-I production and extracellular virus production that we observed for PRV is conserved in HSV-1, which can have important consequences for the rational design of HSV vaccines.

## 2. Materials and Methods

### 2.1. Cells

Vero cells were cultured in Dulbecco’s modified eagle medium (DMEM), supplemented with 10% fetal calf serum (FCS), 100 U/mL penicillin, 0.1 mg/mL streptomycin, and 0.5 mg/mL gentamycin (Life Technologies Europe, Merelbeke, Belgium). Human peripheral blood mononuclear cells (PBMCs) were isolated from the blood of healthy donors using a lymphoprep density gradient and subsequently stored in liquid nitrogen. Blood samples were obtained via the framework agreement between Ghent University and the Red Cross Bloodbank (RKOV_19005).

PBMC were separated on lymphoprep, red blood cells were lysed, washed, resuspended in pDC medium and counted, as described before [7]. PBMCs were cultured in RPMI 1640 (Life technologies), supplemented with 10% fetal calf serum, 100 U/mL penicillin, 0.1 mg/mL streptomycin, 0.5 mg/mL gentamicin, 1 mM nonessential amino acids, 1 mM sodium pyruvate, 2 mM L-glutamine, and 20 μM β-mercaptoethanol (Sigma-Aldrich, St. Louis, MO, USA).

### 2.2. Viruses and Infections

Confluent Vero cells were inoculated with the wild-type (WT) HSV-1 F strain or an isogenic gEnull strain (F-gE-GFP) at a multiplicity of infection (MOI) of 5 in a 96-well plate. Both virus strains were kindly provided by Prof. David C. Johnson (Oregon Health & Science University) and described and thoroughly characterized before [18].

### 2.3. PBMC Co-Incubation with HSV-1-Infected Cells and IFNalpha Production Assays

The PBMC co-incubation and IFNalpha production assays were performed analogous to our earlier PRV-based assays [16]. In brief, at two hours post inoculation (hpi) in a 96-well plate, the inoculum was washed away from confluent mock-inoculated Vero cells or cells inoculated with WT or gEnull HSV-1 (MOI of 5), and 500,000 PBMCs were added per well in a 200 μL pDC medium. After 22 h of co-incubation, supernatant was collected, centrifuged at 500× *g* for 5 min to remove cell debris using an Allegra X-15R centrifuge (Beckman Coulter, Brea, CA, USA), and stored at −80 °C. The IFNalpha concentration was analyzed by ELISA according to the manufacturer’s instructions (pan-IFNalpha ELISA kit, cat nr 3425-1H-6, Mabtech, Nacka Strand, Sweden).

### 2.4. gE Complementation Assays

Vero cells at 80% confluence were transfected with a dual-expression vector plasmid expressing both HSV-1 glycoprotein E and GFP (pBudCE4.1-HSV1-gE-GFP) or with an expression vector plasmid expressing only GFP (pBudCE4.1-GFP). Transfection was performed following the manufacturer’s instructions (Polyplus, Illkirch-Graffenstaden, France). Briefly, cells were transfected in 96-well plates with 0.1 μg plasmid DNA in a 10 μL JetPrime buffer and a 0.2 μL JetPrime reagent per well using a Polyplus JetPrime kit. At 24 h post transfection, Vero cells were inoculated with WT or isogenic gEnull HSV-1 (MOI of 5). At 2 hpi, cells were washed and treated with a low pH medium to inactivate the remaining inoculum virus. Afterward, cells were co-incubated with human PBMCs for 22 h. Supernatant was collected and IFNalpha concentrations were determined via ELISA. Transfection efficiency was tested by flow cytometry (Novocyte, ACEA Biosciences, San Diego, CA, USA) by determining the percentage of GFP-positive cells and was >90%.

For virus titration assays, Vero cells were transfected as explained above. At 24 h post transfection, Vero cells were inoculated with WT or isogenic gEnull HSV-1 (MOI of 5). Afterward, cells were overlaid with a Vero cell medium and further incubated for 14 h or 24 h. Supernatant was collected at 14 or 24 hpi, and extracellular viral titers were determined by titration on Vero cells using a 10-fold serial dilution.

### 2.5. PBMC Stimulation with Cell-Free HSV-1 and IFNalpha Production Assays

PBMC stimulation with cell-free HSV-1 and IFNalpha production assays were performed analogous to our earlier PRV-based assays [7]. In brief, 500,000 PBMCs were co-incubated with either equal amounts of WT HSV-1 or isogenic gEnull HSV-1 cell-free virus (2.5 × 10^6^ TCID_50_ infectious virus) for 22 h. After co-incubation, supernatant was collected, centrifuged at 500× *g* for 5 min to remove cell debris using an Allegra X-15R centrifuge (Beckman Coulter, Brea, CA, USA), and stored at −80 °C. The IFNalpha concentration was analyzed by ELISA according to the manufacturer’s instructions (pan-IFNalpha ELISA kit, cat nr 3425-1H-6, Mabtech, Nacka Strand, Sweden).

### 2.6. PBMC Stimulation with Supernatant of HSV-1-Infected Cells and IFNalpha Production Assays

Confluent Vero cells were mock-inoculated or inoculated with WT HSV-1 F strain or an isogenic gEnull strain in a 96-well plate at an MOI of 5. At 2 hpi, the inoculum was washed away, and a citrate buffer (40 mM sodium citrate, 10 mM KCl, 135 mM NaCl (pH 3)) was added to the cell surface for 2 min to inactivate non-entered virions. Afterward, cells were washed, covered with a Vero cell medium, and incubated further at 37 °C for either 14 or 24 h. For the time-course assay, supernatant was collected between 8 and 24 hpi at 4 h increments. Supernatant was centrifuged at 500× *g* for 5 min to remove cell debris using an Allegra X-15R centrifuge (Beckman Coulter) and stored at −80 °C. Next, 500,000 PBMCs were co-incubated with 200 μL supernatant in a 96-well plate for 22 h. After co-incubation, supernatant was collected, centrifuged at 500× *g* for 5 min to remove cell debris using an Allegra X-15R centrifuge (Beckman Coulter, Brea, CA, USA), and stored at −80 °C. The IFNalpha concentration was analyzed by ELISA according to the manufacturer’s instructions (pan-IFNalpha ELISA kit, cat nr 3425-1H-6, Mabtech, Nacka Strand, Sweden).

### 2.7. IFNalpha Production by PBMC upon Stimulation with WT HSV-1-Infected Cells in the Presence of Virus-Free Supernatant of gEnull HSV-1- or WT HSV-1-Infected Cells

Confluent Vero cells were mock-inoculated or inoculated with WT HSV-1 F strain or an isogenic gEnull strain in a 96-well plate at an MOI of 5. At 2 hpi, the inoculum was washed away, and a citrate buffer (40 mM sodium citrate, 10 mM KCl, 135 mM NaCl (pH 3)) was added for 2 min to inactivate non-entered virions. Afterward, cells were washed, covered with a Vero cell medium, and incubated further at 37 °C for 14 h. Supernatant was collected, and cell debris was removed at 1000 g for 10 min using an Allegra X-15R centrifuge (Beckman Coulter). Supernatant was ultracentrifuged to remove viral particles at 100,000× *g* for 4 h (=“virus free supernatant”) using a SW41Ti swinging bucket rotor in an Optima XE ultracentrifuge (Beckman Coulter, Brea, CA, USA). Next, confluent Vero cells were inoculated with WT HSV-1 (MOI of 5). At 2 hpi, the inoculum was washed away, and a citrate buffer (40 mM sodium citrate, 10 mM KCl, 135 mM NaCl (pH 3)) was added for 2 min to inactivate non-entered virions. Subsequently, infected cells were overlaid with 200 μL virus-free supernatant from either WT HSV-1- or gEnull HSV-1-infected cells, and 500,000 PBMCs were added. After 22 h of incubation, supernatants were collected, and IFNalpha concentrations were determined by ELISA according to the manufacturer’s instructions (pan-IFNalpha ELISA kit, cat nr 3425-1H-6, Mabtech, Nacka Strand, Sweden).

### 2.8. Extracellular Virus Titers

Confluent Vero cells were inoculated with WT HSV-1 or isogenic gEnull HSV-1 in a 96-well plate at an MOI of 5. At 2 hpi, the inoculum was washed away, and a citrate buffer (40 mM sodium citrate, 10 mM KCl, 135 mM NaCl (pH 3)) was added to the cell surface for 2 min to inactivate non-entered virions. Afterward, cells were washed, covered with a Vero cell medium, and incubated further at 37 °C. At different time points, a cell medium was collected, centrifuged at 500× *g* for 5 min to remove cell debris using an Allegra X-15R centrifuge (Beckman Coulter), and stored at −80 °C. Viral titers in the supernatant were analyzed by titration on Vero cells using a 10-fold serial dilution.

### 2.9. Statistical Analysis

Statistical analysis was performed using GraphPad Prism. IFNalpha data were analyzed using Student’s *t*-test or the repeated-measures analysis of variance (ANOVA) at the 5% significance level; post hoc comparisons between different conditions were performed by Tukey’s range test. Log_10_ virus titers were analyzed by Student’s *t* test at the 5% significance level.

## 3. Results

### 3.1. Lack of gE Results in Increased HSV-1-Induced IFNalpha Production by Human Peripheral Blood Mononuclear Cells

**Figure 1:**
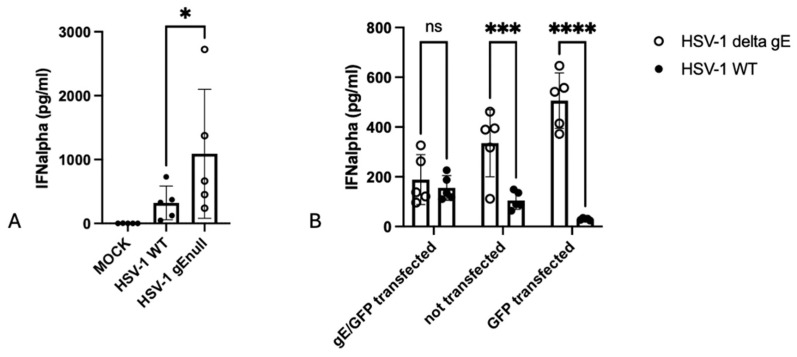
Vero cells infected with gEnull HSV-1 trigger increased IFNalpha by PBMCs compared with cells infected with WT HSV-1. (**A**) HSV-1 gEnull-infected Vero cells trigger an increased IFN-I response in human PBMCs compared with cells infected with isogenic WT HSV-1. Vero cells were mock-inoculated or inoculated with WT or gEnull HSV-1 strains. At 2 hpi, cells were washed and co-incubated with human PBMCs for 22 h. IFNalpha concentrations in the supernatant were determined by ELISA. (**B**) Vero cells were either transfected with a plasmid expressing both HSV-1 gE and GFP, not transfected or transfected with a plasmid expressing only GFP. At 24 h post transfection, Vero cells were inoculated with WT or isogenic gEnull HSV-1. The inoculated cells were co-incubated with human PBMCs for 22 h. Supernatant was collected, and IFNalpha concentrations were determined by ELISA. Graphs show means, standard deviations, and individual data points of 5 independent repeats. (**A**,**B**): ns, non significant; *, *p* < 0.05; ***, *p* < 0.001; ****, *p* < 0.0001 using two-way ANOVA.

Production of IFNalpha by human or porcine peripheral blood mononuclear cells (PBMCs) in response to HSV-1 or PRV, respectively, has been shown to be virtually exclusively caused by pDCs [7,17,19]. Hence, to analyze whether, like for PRV [16,17], the lack of gE in HSV-1 may result in increased IFN-I production by pDCs/PBMCs, human PBMCs were co-incubated for 22 h with Vero cells infected with WT or isogenic gEnull HSV-1 from 2 hpi onward. Figure 1A shows that, in line with what we observed earlier for PRV, cells infected with gEnull HSV-1 triggered significantly increased IFNalpha production by PBMCs compared with cells infected with WT HSV-1. To make sure that the observed phenotype is caused by the absence of gE in the gEnull mutant, a gE complementation assay was conducted. To this end, Vero cells were transfected with an HSV-1 gE-encoding expression vector. At 24 h post transfection, the cells were infected with WT or isogenic gEnull HSV-1 (MOI of 5) and co-incubated with human PBMCs for 22 h. IFNalpha concentration in the supernatant was determined by ELISA. Transfection with glycoprotein E completely abolished the increased IFNalpha response by gEnull HSV-1 (Figure 1B).

### 3.2. Lack of gE Results in Increased Extracellular Virus Titers in HSV-1-Infected Vero Cells

**Figure 2:**
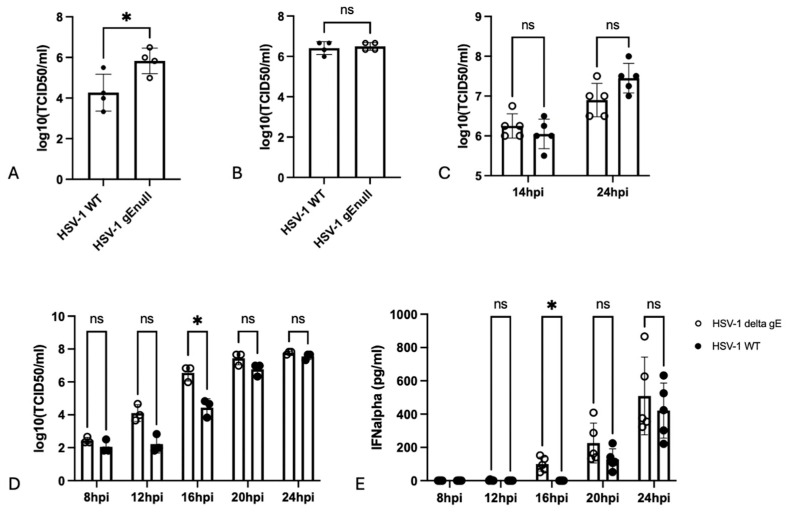
Infection of Vero cells with gEnull HSV-1 results in increased virus titers in the supernatant at relatively early in infection compared with WT HSV-1. (**A**,**B**) Vero cells infected with gEnull HSV-1 display higher extracellular virus titers compared with cells infected with WT HSV-1 at 14 hpi (**A**). At 24 hpi (**B**) HSV-1 virus titers of WT and gEnull HSV-1 are comparable. (**C**) Vero cells were transfected with a gE-expressing plasmid. Twenty-four hours later, cells were inoculated with WT or isogenic gEnull HSV-1. Supernatants were collected at 14 or 24 hpi, and extracellular viral titers were determined. Graphs show means, standard deviations, and individual data points of 4 independent repeats (**A**–**C**): ns, not significant; *, *p* < 0.05, Student’s *t*-test. (**D**) Vero cells were inoculated with WT or gEnull HSV-1, and extracellular viral titers in the supernatant were determined at different time points. (**E**) Vero cells were inoculated with WT or gEnull HSV-1. At different time points, supernatant was collected and co-incubated with human PBMCs for 24 h. IFNalpha concentrations were determined by ELISA. Graphs show means, standard deviations, and individual data points of 5 independent repeats (**D**,**E**): ns, not significant; *, *p* < 0.05, two-way ANOVA with Šidák correction for multiple comparisons.

In PRV, we reported that the increased IFNalpha production by PBMCs/pDCs in response to gEnull PRV-infected cells compared with WT PRV-infected cells correlates with an increased production of pDC-activating extracellular virus particles at relatively early time points post infection with gEnull PRV compared with WT PRV [16]. To assess whether a similar correlation can be established in HSV-1, extracellular virus titers were determined at different time points post inoculation (14 and 24 hpi) of Vero cells with WT or gEnull HSV-1. Figure 2A,B show that, like in PRV, extracellular titers of gEnull HSV-1-infected Vero cells are higher than those of WT HSV-1-infected cells at a relatively early time point post inoculation (14 hpi), whereas extracellular titers of both viruses are comparable late in infection (24 hpi). Again, a gE-complementation assay was performed to confirm that the observed phenotype is gE-mediated. To this end, gE-transfected Vero cells were inoculated at 24 h post transfection with WT or gEnull HSV-1. At 14 and 24 hpi, supernatant was collected, and virus titers were determined. As shown in Figure 2C, both at 14 and 24 hpi, virus titers of WT and gEnull HSV-1 were comparable in gE-transfected Vero cells.

Next, more extensive time-course assays were performed, which confirmed the previous results (Figure 2D,E). These assays showed that the increased extracellular titer for gEnull HSV-1 can already be weakly observed at 8 hpi, increased at 12 hpi, reached statistical significance at 16 hpi, decreased again at 20 hpi, and disappeared at 24 hpi (Figure 2C). This pattern corresponded with the IFNalpha production levels by PBMCs, with a significantly stronger response for gEnull HSV-1 at 16 hpi, whereas this increased response for gEnull HSV-1 was less prominent at 20 hpi and particularly 24 hpi (Figure 2D). Supernatants harvested at 8 and 12 hpi did not trigger detectable IFNalpha by PBMC, likely because the amount of virus in the supernatant did not yet reach the threshold to trigger IFNalpha production by PBMCs.

### 3.3. Stimulation of PBMC with Equal Amounts of HSV-1 WT or HSV-1 gEnull Virions Results in Similar IFNalpha Production

Our data suggest that the increased IFNalpha production by human PBMCs in response to gEnull HSV-1-infected Vero cells may be caused by the earlier production of extracellular virus in gEnull HSV-1-infected Vero cells. However, different alternative and/or complementary explanations can be given to explain the increased IFNalpha production by PBMCs in response to gEnull HSV-1-infected Vero cells.

**Figure 3:**
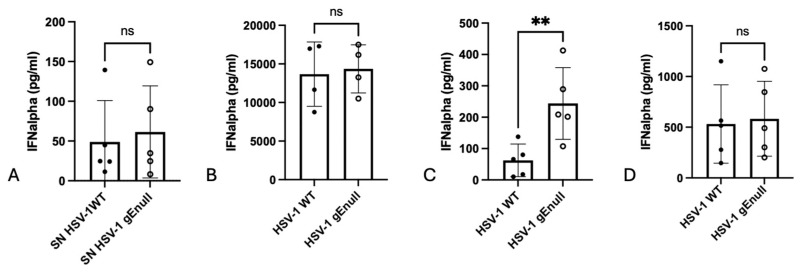
Equal amounts of WT or gEnull HSV-1 trigger similar IFNalpha production by PBMCs. (**A**) Supernatant of WT HSV-1- or gEnull HSV-1-infected Vero cells was collected at 14 hpi and ultracentrifuged to remove viral particles. Next, WT HSV-1 inoculated Vero cells were overlaid with virus-free supernatant of either WT or gEnull HSV-1-infected cells and co-incubated with human PBMCs. After 22 h, IFNalpha concentrations in the supernatant were determined. (**B**) Human PBMCs were incubated with equal amounts of WT or gEnull HSV-1 infectious virus particles (2.5 × 10^6^ TCID_50_) for 24 h. Afterward, IFNalpha concentration in the supernatant was determined by ELISA. (**C**,**D**) Vero cells were inoculated with WT or gEnull HSV-1 and incubated for 14 h (**C**) or 22 h (**D**). Supernatant was collected, and human PBMCs were incubated with the prepared supernatant for 24 h. IFNalpha concentrations were determined. Graphs show means, standard deviations, and individual data points of 5 independent repeats (**A**–**D**): ns, not significant; **, *p* < 0.01, using ratio paired Student’s *t*-test.

First, an alternative explanation could be that the supernatant of gEnull HSV-1-infected Vero cells contains (increased/decreased levels of) non-virus-related factors, such as cytokines, that may affect the IFNalpha response by PBMCs to HSV-1-infected cells. To assess this, an additional assay was designed. In this assay, supernatants were collected from WT HSV-1- and gEnull HSV-1-infected cells at 14 hpi, a time point where the increase in gEnull HSV-1 virus production and IFNalpha production by PBMCs is evident (Figure 1A and Figure 2A). Next, supernatants were cleared of virus via ultracentrifugation. These virus-cleared supernatants were then added to WT HSV-1-infected Vero cells and PBMCs were added, and the resulting IFNalpha response was analyzed by ELISA. This assay showed that the virus-cleared supernatant of gEnull HSV-1-infected Vero cells does not show an increased potency to trigger PBMC-mediated IFNalpha production compared with the virus-cleared supernatant of WT HSV-1-infected Vero cells (Figure 3A), further supporting the notion that the increased extracellular virus production in gEnull HSV-1-infected Vero cells compared with WT HSV-1-infected cells is responsible for the increased IFNalpha production by PBMCs.

Second, it is possible that gEnull HSV-1 virions have an increased ability to trigger pDC/PBMC activation, thereby eliciting an increased IFNalpha response. To assess this possibility, PBMCs were stimulated with equal TCID_50_ of cell-free WT or gEnull HSV-1 virions for 22 h. The resulting IFNalpha concentrations showed that no significant differences in IFNalpha response between both viruses can be observed (Figure 3B).

Third, it is possible that the presence/absence of the gE glycoprotein on the plasma membrane of WT/gEnull HSV-1-infected Vero cells may affect the ability of PBMCs/pDCs to produce IFNalpha, e.g., via ligand–receptor interactions. Therefore, to assess whether or not the increased IFNalpha response by PBMCs is independent of the infected cell (surface), the supernatant of Vero cells infected with WT or gEnull was collected at 14 or 24 hpi and added to PBMCs for 24 h, followed by the analysis of IFNalpha production by ELISA. In line with the higher extracellular virus titer of gEnull HSV-1 at 14 hpi compared with that of WT HSV-1 (Figure 2A), stimulation of PBMCs with the supernatant collected at 14 hpi from gEnull HSV-1-infected Vero cells induced a higher IFNalpha response compared with the supernatant of WT HSV-1-infected Vero cells (Figure 3C). In line with the comparable extracellular virus titers between both viruses at 24 hpi (Figure 2B), supernatants collected at 24 hpi of Vero cells infected with either WT or gEnull HSV-1 triggered similar IFNalpha responses (Figure 3D).

Together, these assays confirm that the increased IFNalpha response by PBMCs in response to gEnull HSV-1-infected Vero cells compared with WT HSV-1-infected cells can be attributed to the increased amount of extracellular virions produced relatively early in infection of Vero cells.

## 4. Discussion

HSV and PRV are both alphaherpesviruses, with humans and pigs as their respective natural host. Previous research has shown that epithelial cells infected with the attenuated PRV strain Bartha, the most successful PRV vaccine worldwide [15], trigger hyperactivation of pDCs, with a substantial increase in IFNalpha production compared with cells infected with wild-type (WT) PRV [16]. The Bartha vaccine strain genome contains a deletion in the unique short region that includes the US8 gene, encoding the viral gE glycoprotein. This lack of gE was found to result in a rapid rise in extracellular titers in infected epithelial cells, which, in turn, resulted in a hyperactivation of pDCs and an increased production of IFNalpha [16,17]. The current report shows that this phenotype appears to be conserved in HSV-1. The data demonstrate that epithelial Vero cells infected with HSV-1 that lacks the expression of gE trigger increased IFNalpha responses by peripheral blood mononuclear cells (PBMCs). As in PRV, we found that the HSV-1 gEnull strain induced an earlier increase in extracellular virus titers in infected Vero epithelial cells compared with the WT strain. Additional assays confirmed that the observed increase in IFNalpha production by PBMCs by gEnull HSV-1-infected Vero cells cannot be attributed to an IFNalpha-suppressing effect of the glycoprotein gE on the infected cell surface or to an increased ability of gEnull HSV-1 virions to trigger IFNalpha production by PBMCs.

Our data contribute to the notion that several functions associated with the viral gE glycoprotein appear to be conserved across different alphaherpesviruses like HSV-1 and PRV, such as its role in anterograde axonal transport [20,21,22,23,24] and its ability to exert Fc receptor activity [25,26,27,28,29]. Hence, our current data underscore that the functional palette of this important virulence factor is substantially conserved at least in these two viruses.

The exact reason why titers in the supernatant of epithelial cells infected with gEnull HSV-1 or gEnull PRV increase more rapidly compared with those of their WT counterparts remains to be elucidated. Previous research has shown that, in HSV-1, gE has a function in sorting viral particles toward basolateral cell junctions in polarized epithelial cells, possibly by interacting with the AP-1 sorting adaptor protein [30]. Deletion of gE interfered with this process, and the virus was increasingly transported to the apical side of the cells and released in the supernatant, resulting in a more rapid increase in the extracellular titers of the gEnull virus in polarized cells [30]. Interestingly, in line with our current results, other groups reported increased extracellular virus titers of gEnull HSV-1 compared with WT HSV-1 in Vero cells [31,32], although this was not connected to an impact on antiviral IFNalpha production, as described in the current report. Overall, it is assumed that the lack of gE impairs the coordinated viral cell-to-cell spread, thereby promoting the release of virions in the extracellular milieu. We presume that the reason why, at late stages of infection (24 hpi), we did not observe a difference in the extracellular virus titer between WT HSV-1- and gEnull HSV-1-infected Vero cells may be that, at these late stages of infection, intracellular sorting mechanisms in infected epithelial cells may be impaired and/or cells may start to succumb to the infection, releasing accumulated intracellular virus into the extracellular environment. Our current report indicates that the rapid release of virions in the extracellular environment of the gEnull strain may come at the expense of increased immune cell-mediated antiviral IFNalpha responses. It is interesting to note that cell culture adaptation of PRV by extensive cell culture passaging often leads to the deletion of gE, which, for example, has occurred during the cell-culture-based generation of commonly used attenuated PRV vaccine strains Bartha and Bucharest [16]. Although speculative, it can be hypothesized that the combined lack of the complex host neuronal cell type (requiring long-distance axonal spread) and immune effectors during cell culture passage steers the genetic adaptation of the virus toward a more simple variant that lacks the gE gene. The lack of gE contributes to higher extracellular virus titers, which may be beneficial in cell culture, but comes at the expense of impaired neuronal spread and increased immune cell-mediated IFN-I responses, which are interesting features for vaccine candidates.

Upon alphaherpesvirus infection, both heavy and light particles (L-particles) are produced. Heavy particles are DNA containing infectious virus particles; L-particles do not contain a capsid or DNA and are non-infectious [33]. Only heavy particles trigger pDC activation and IFN-I production, while L-particles do not, likely because they lack DNA [7]. We showed earlier that, both in HSV-1 and PRV, L-particles can partially suppress pDC activation triggered by the infectious virus [7]. Moreover, other immune-modulating properties were reported before for L-particles of HSV-1, such as CD83 downregulation on dendritic cells [34,35] There is evidence that the particle-to-PFU ratio at 18 hpi in the supernatant of Vero cells infected with WT HSV-1 is somewhat higher (particle/PFU ratio 6) compared with that of gEnull HSV-1 (particle/PFU ratio 4) [36]. Although this difference is very small, it is possible that this may reflect a slightly higher level of L-particles in the supernatant of WT HSV-1-infected cells compared with that in gEnull HSV-1-infected cells. Since we showed before that L-particles of HSV-1 (or PRV) suppress the interferon response by pDCs [7], we cannot formally exclude that this, to some extent, may also contribute to the observed gE-dependent differences in interferon response observed in the current study. However, we showed that stimulation of PBMCs with supernatants of WT HSV-1- or gEnull HSV-1-infected Vero cells that contained equal amounts (2.5 × 10^6^ TCID_50_) of infectious virus triggered similar IFNalpha responses (Figure 3B). If the different particle-to-PFU ratio would contribute to the observed differences in IFNalpha production by PBMCs in response to gEnull versus WT HSV-1, one would also expect different IFNalpha levels in this experimental setup, which was not the case. Nonetheless, it may be worthwhile to further assess if shifts in particle/PFU ratios may lead to different IFNalpha production by PBMCs.

As mentioned before, pDCs are, despite their low numbers in blood, the main IFN-I-producing cell type in the body [5]. They can produce up to 1000 times more IFN-I than any other cell type [37]. Previous studies have shown that the IFN-I response of PBMCs to HSV-1 is virtually entirely caused by the pDC population in a TLR-9-dependent manner [19]. We obtained similar results for porcine pDCs/PBMCs in response to the porcine alphaherpesvirus PRV [7,17]. This potent IFN-I-producing ability of pDCs makes them a crucial player not only in the innate defense against viral infections [38] but also more generally in creating an environment that suppresses viral replication. Indeed, both in humans and pigs, pDC-mediated IFN-I production is important to boost cytotoxic natural killer cells (NK cells), stimulate maturation of dendritic cells, and, importantly, steer the adaptive immune response toward a Th1-dominated immune response and expansion of memory T-cell populations, thus establishing long-lasting (antiviral) immunity and representing an important target in virus vaccine design [12,13,19,39]. As a consequence, stronger vaccine-induced activation of pDCs can result in a more qualitative and effective adaptive immune response [40]. The current data indicate that gEnull HSV-1 infection of Vero cells leads to a more rapid increase in extracellular virus titers, resulting in a faster and stronger activation of PBMCs to produce IFNalpha, resulting, in turn, in increased levels of this antiviral cytokine. Although it is obviously difficult and speculative to translate in vitro findings to in vivo situations, these data may possibly have important consequences in vivo, since a faster and increased production of IFNalpha by PBMCs not only may result in increased direct antiviral activities by triggering an antiviral state in non-infected cells but also may lead to an increased stimulation of other aspects of the antiviral immune response, including a Th1 response that is highly desired in the response against herpesviruses [12,13,14]. Our data thereby contribute to the idea that deletion of gE may represent a potentially important aspect of rational vaccine design against HSV-1.

## Data Availability

Data are contained within the article.
